# Discovering human cell‐compatible gene therapy virus variants via optimized screening in mouse models

**DOI:** 10.1111/cpr.13565

**Published:** 2023-10-20

**Authors:** Moyu Dai, Ning Yang, Kai Xu, Jingwen Zhang, Xueke Li, Ying Zhang, Wei Li

**Affiliations:** ^1^ State Key Laboratory of Stem Cell and Reproductive Biology, Institute of Zoology Chinese Academy of Sciences Beijing China; ^2^ Institute for Stem Cell and Regenerative Medicine Chinese Academy of Sciences Beijing China; ^3^ University of Chinese Academy of Sciences Beijing China; ^4^ Beijing Institute for Stem Cell and Regenerative Medicine Chinese Academy of Sciences Beijing China; ^5^ College of Life Science Nankai University Tianjin China

## Abstract

In gene therapy, intravenous injection of viral vectors reigns as the primary administration route. These vectors include adeno‐associated viruses, adenoviruses, herpes viruses, rhabdoviruses and others. However, these naturally occurring viruses lack inherent tissue or organ tropism for tailored disease treatment. To address this, we devised an optimized process involving directed viral capsid evolution, organ‐specific humanized mouse models and in vitro‐in vivo virus screening. Our approach allows for the rapid generation specifically modified adeno‐associated virus variants, surpassing the time required for natural evolution, which spans millions of years. Notably, these variants exhibit robust targeting of the liver, favouring chimeric human liver cells over murine hepatocytes. Furthermore, certain variants achieve augmented targeting with reduced off‐target organ infection, thereby mitigating dosage requirements and enhancing safety in gene therapy.

## Graphical abstract

The naturally occurring viruses lack inherent tissue or organ tropism for tailored disease treatment. In this study, we devised an optimized process involving oligonucleotide pool, screening on RNA level, organ‐specific humanized mouse models and in vitro*‐*in vivo validation strategies. We employed site‐directed insertion, incorporating millions of non‐naturally occurring peptides into a specific capsid position and rapidly generated new variants, some of which exhibited robust targeting of the liver, favouring chimeric human liver cells over murine hepatocytes. We characterized novel variants derived from AAV9 by the optimized screening approach, contributing to gene and cell therapy, cell fate determination, reversing senility and regenerative medicine research.

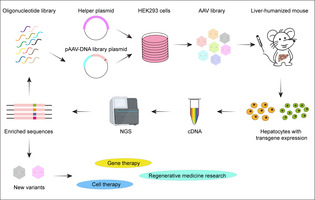

## 1 INTRODUCTION

Gene therapy, originally conceived as a method to treat genetic diseases by supplying functional genes to address specific mutations,^1^ has demonstrated a pivotal role in clinical treatment. For example, in patients with spinal muscular atrophy, gene therapy allows individuals with a life expectancy of less than 2 years to survive without constant respiratory support and stand with assistance.^2^, ^3^ In recent years, remarkable advancements in our ability to manipulate DNA sequences and control cellular fate have propelled gene therapy beyond conventional bounds in human or animal models. This progress enables precise and persistent correction of genetic defects,^4^ targeted epigenetic modifications,^5^ directed cell reprogramming,^6^, ^7^, ^8^, ^9^ cellular aging reversal,^10^, ^11^, ^12^, ^13^ and even lifespan extension.^14^, ^15^, ^16^ These advances strongly indicate that gene therapy will play an increasingly crucial role in medicine going forward.

Among a series of vectors, adeno‐associated virus (AAV) currently holds the leading position in clinical gene therapy, credited to its low pathogenicity and immunogenicity, as well as its broad tropism and high stability.^17^, ^18^ Efforts to engineer the AAV capsid have been ongoing since its remarkable potential in gene therapy was discovered,^19^, ^20^ employing techniques such as rational design, DNA shuffling, directed evolution and in silico approaches. However, these approaches face limitations due to the need for a prior understanding of the specific viral entry mechanism and the affinity of the original viral sequence. Importantly, the extensive and costly research process poses challenges in meeting the continuous improvement requirements for the AAV capsid, which are crucial for achieving the ideal state of a gene therapy vector characterized by enhanced and specific transduction of the target organ, reduced intravenous dosage requirement and minimized immune risks.

Among all AAV variants, adeno‐associated virus 9 (AAV9) stands out for its highest infectivity and broadest tropism,^21^ making it the preferred choice for gene therapy in systemic diseases affecting the central nervous system (CNS), liver, muscle and more, as exemplified by its application in the treatment of SMA.^2^ However, the broad tropism of AAV9 also poses a significant challenge due to its lack of organ specificity. Higher doses of AAV9 are often required to achieve sufficient infection and therapeutic effects in targeted organs, potentially leading to enhanced infection in non‐targeted organs and heightened immune risks.^22^, ^23^ This issue was highlighted in a trial for children with X‐linked myotubular myopathy (MTM), where high doses resulted in severe hepatotoxicity, proving fatal for two patients due to an immune response.^24^

The liver, crucial in gene therapy, has been the target of approximately 30% of AAV‐based clinical trials.^25^ Hepatocytes are the most common type of liver cell and the actual target of gene therapy.^26^ This study aimed to demonstrate the effectiveness of an optimized approach by screening for AAV9 capsid variants with enhanced human hepatocyte infectivity both in vitro and in vivo. To achieve this, we employed site‐directed insertion, incorporating millions of non‐naturally occurring peptides into a specific capsid position. The goal was to rapidly generate new variants practically packaged into recombinant viral particles capable of infecting human liver cells. Additionally, from this extensive pool of candidates, we expected to identify variants exhibiting enhanced hepatocyte infectivity while reducing infection in non‐targeted organs.

## 2 MATERIALS AND METHODS

### 2.1 Plasmid construction

The transgene plasmid pAAV‐CMV‐luciferase‐enhanced green fluorescent protein (EGFP) was constructed by isolating the backbone from the pX602 plasmid (Addgene, 61593) and synthesizing the sequence of CMV‐luciferase‐T2A‐EGFP‐polyA from GenScript Biotech Corporation. Transgene plasmids with barcodes were constructed by inserting a specific barcode sequence between EGFP and polyA in pAAV‐CMV‐luciferase‐EGFP. The rAAV‐Cap‐in‐cis genome plasmid contains an EGFP expression cassette, the AAV9 capsid gene and regulatory sequences,^27^, ^28^ and the backbone from the pX602 plasmid (Addgene, 61593). Helper plasmids of the variants were generated by inserting fragments with enriched sequences into the accessory capsid plasmid pDP9 (Plasmid Factory, PF0439). We also modified the pDP9 plasmid to eliminate capsid protein expression by inserting an in‐frame stop codon in the reading frames of each capsid protein VP1‐VP3, generating the helper plasmid pRep‐AAP for library production.^29^

### 2.2 AAV plasmid library generation

Using the 7 × NNK saturation mutagenesis strategy, we designed a randomized (21‐base) heptamer insertion using degenerate primers containing mixed bases. N can be an A, C, G, or T base and K can be G or T. We synthesized a primer with a randomized (21‐base) heptamer insertion and amplified the library fragment with Q5 High‐Fidelity 2X Master Mix (NEB, M0494S) and primers (5′‐CTTTAATTTTT‐GGCAAACAAGGTACCGGAAGAGACAACGTGGATGCG‐3′ and 5′‐ATTCCTTGGTTTTGAACCCAACCGGTCTGCGCCTGTGCMNNMNNMNNMNNMNNMNNMNNTTGGGCACTCTGGTGGTTTGTG‐3′). For the random 7‐mer insert capsid library constructs, KpnI (NEB, R3142S) and AgeI (NEB, R3552L) were used to remove sequences between amino acids 546 and 599 in VP1 of rAAV‐Cap‐in‐cis genome plasmid. After digestion, the cut skeletons were purified using Zymoclean Gel DNA Recovery Kit (ZYMO RESEARCH, D4008). The NEBuilder HiFi DNA Assembly Master Mix (NEB, E2621) was used to insert the library fragment into the linearized skeleton. We synthesized more than 8000 capsid variants based on the enrichment score of the first round via oligonucleotide pools. The second round of plasmid library construction and extraction were the same as those of the first round.

### 2.3 Cell culture

HEK293 (Procell, CL‐0001), Huh7 (Procell, CL‐0120) and HepG2 (ATCC, HB‐8065) cells were cultured in Dulbecco's modified Eagle's medium (DMEM) (Gibco, C11995500BT) supplemented with 10% foetal bovine serum (FBS) (Gibco, 10099141) and 1% penicillin‐streptomycin (Gibco, 15140‐122). Primary human hepatocytes (PHHs) were provided by Celsis In Vitro Technologies (Baltimore, MD, USA), thawed, and cultured according to manufacturer's instructions.

### 2.4 Animals and transplantation

The Institutional Animal Care and Use Committee of the Institute of Zoology, Chinese Academy of Sciences (IOZ, CAS) approved all animal experiments (reference number: IOZ‐IACUC‐2022‐167). All Tet‐uPA^Tg^ Rag2^null^ Il2rg^null^ (URG) mice were purchased from Beijing Vitalstar Biotechnology Co., Ltd. and raised in IOZ, CAS animal facilities. Cryopreserved PHHs were suspended in DMEM supplemented with 10% FBS, and trypan blue quantified cell viability (typically >90%). PHHs (1 × 10^6^ cells/animal) were injected into spleens of mice.^30^ Blood samples were collected and human albumin was quantified using the Human Albumin ELISA Quantitation kit (Bethyl, E88‐129). Humanized‐URG mice (Hu‐URG) with 2–2.5 mg/mL human albumin achieved approximately 40%–50% human hepatocyte repopulation.^31^ All virus injections were administered intravenously by tail.

### 2.5 In vivo selection of directed evolution

AAV vectors were intravenously administered to Hu‐URG mice. Two weeks later, mice were anaesthetised by saline perfusion, and livers were harvested and further digested into single‐cell suspension for Fluorescence‐Activated Cell Sorting (FACS). EGFP‐positive (EGFP^+^) hepatocytes were sorted and total RNA was extracted using TRIzol (Invitrogen, 15596018). The mRNA was enriched from total RNA samples with PureLink® RNA Mini Kit (Invitrogen, 12183018A) and treated with RNase‐Free DNaseI (TIANGEN, RT411) according to the manufacturer's instructions. Subsequently, cDNA was synthesized using M‐MLV Reverse Transcriptase (Promega, M1705) and random primers (Promega, C1181). Capsid variant sequences were amplified with Q5 High‐Fidelity 2X master mix (NEB, M0494S) and primers flanking the 7‐mer insert (5′‐TAACCCGGTAGCAACGG‐AGTCCTATG‐3′ and 5′‐TGCCAAACCATACCCGG‐AAGTATTCC‐3′), which added Illumina adaptors and unique indices. Amplicons were pooled at an equimolar ratio and sequenced using an Illumina NextSeq system.

### 2.6 In vivo and in vitro characterization of AAV vectors

For in vivo characterization, three male Hu‐URG mice were injected with 2.0 × 10^12^ vg of rAAV mix via intravenous administration. After 2 weeks, mice were euthanized, and their livers were digested for FACS. Additionally, we collected muscular tissue, heart, lung and kidney for DNA and RNA extraction. For in vitro characterization, PHHs were infected with the rAAV mix at multiplicities of infection (MOIs) of 1 × 10^4^ and 1 × 10^5^. After transduction for 48 hours, cells were collected for DNA and RNA extraction. Total RNA was extracted and treated as described above, and DNA was isolated using the E.Z.N.A. MicroElute Genomic DNA Kit (Omega, D3096‐02) according to the manufacturer's instructions. DNA and cDNA were amplified with Q5 High‐Fidelity 2X Master Mix and primers flanking the barcode region (5′‐CCTGAGCAAAGACCCCAACGAG‐3′ and 5′‐GCTGCAATAAACAAGTTGGGGTG‐3′). Amplicons with Illumina adapters and unique indices were pooled at equimolar ratios and sequenced on an Illumina NextSeq platform.

### 2.7 Next generation sequencing analysis

The raw fastq files were aligned to the AAV9 template DNA fragment, which contained a 6‐bp sequence flanking the insertion site. All the diversified 21‐bp variant sequences and read counts (RCs) were extracted. A custom script was used to normalize the aligned data and translate the DNA sequence into an amino acid sequence. Variants were counted in each sample and normalized to the sequencing depth of the run to assign each variant a reads per million (RPM) score. Variants were ranked according to the ratio of variant RPM in the sample to variant RPM in the matched sequenced virus library sample, to account for the unequal distribution of variants in the injected virus library. For the pooled rAAV characterization experiment, the enrichment score of each barcode sequence represented the corresponding variant. Heatmap analysis was performed with the ‘pheatmap’ function in R. Frequency distribution maps were generated using the R ‘ggseqlogo’ function. For empirical cumulative distribution frequency (ECDF) analysis, custom‐made scripts were used from Reference 32.

### 2.8 Detection of transduction efficiency in multiple cell lines

Before transduction, the cells were seeded into a 96‐well plate (Corning Mediatech, 3903) at a density of 2 × 10^4^ cells/well. The AAVs were transduced at MOIs of 1 × 10^4^ and 1 × 10^5^. After 72 h, the luciferase activity was measured using the Bright‐Lite Luciferase Assay System (Vazyme, DD1204) according to the manufacturer's instructions.

### 2.9 Statistical analysis

The experimental data were statistically analysed using GraphPad Prism 8 software. Significant differences between samples were analysed using the unpaired Student's *t*‐test and one‐way analysis of variance (ANOVA) using Dunnett's multiple comparison test.

### 2.10 Software and algorithms

Computational modelling of the VR‐VIII loop of AAV‐L8 was performed by SWISS‐MODEL server, and all structures were visualized in ChimeraX. All image processing was performed using Adobe Illustrator CC, R package ‘ggseqlogo’, R package ‘pheatmap’ and custom‐made scripts in R. For data analysis, Microsoft Excel 2016 and GraphPad Prism 8 were used.

## 3 RESULTS

### 3.1 Deriving millions of VP3 variants of AAV9 through site‐specific insertion

**FIGURE 1:**
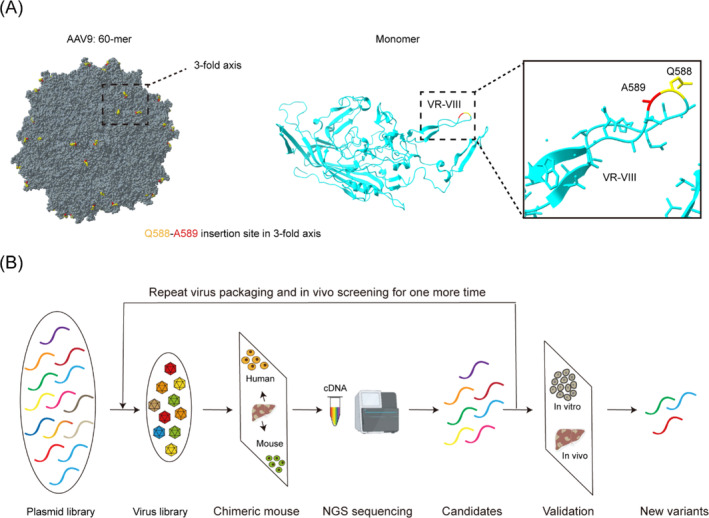
Capsid engineering locations and workflow. (A) Structural model of the AAV9 capsid (PDB 3UX1) with the insertion site for the 7‐mer capsid library in the 60‐meric (left) and monomeric (middle) forms by ChimeraX. The insertion location is magnified in the black rectangle frame (right). (B) Screening and validation workflow of AAV9 capsid variants with hepatocyte tropism.

According to the results of structure resolution,^33^ AAV is a small compact protein particle with a diameter of 26 nm and a regular icosahedral structure. For both native and recombinant AAV, the capsid of AAV particles is composed of three homologous proteins, VP1, VP2 and VP3. VP2 and VP3 are N‐terminal truncated versions of VP1.^34^ As the most abundant capsid subunit, VP3, comprises a major part of the capsid surface and plays a crucial role in the process of virus‐cell contact, mediating receptor or heparin‐dependent viral endocytosis.^35^ The structure of VP3 contains nine highly variable extended loops called variable regions (VR‐I to VR‐IX), which are predicted to be less critical for capsid assembly, but essential for determining cell tropism and antigenicity.^33^, ^36^ Modifications of the VR‐VIII, which is located at the top of the 3‐fold axis of the AAV capsid protein, can change the infection ability of AAV on cells.^33^ Therefore, we theorized that introducing diversity into the amino acid 588 would enhance the transduction of human hepatocytes (Figure 1A). By DNA recombination, the synthesized oligo library can be used to guide the in‐vivo directed evolution of AAV9 variants, and new mutants can be screened out using next generation sequencing technology (Figure 1B).

**FIGURE 2:**
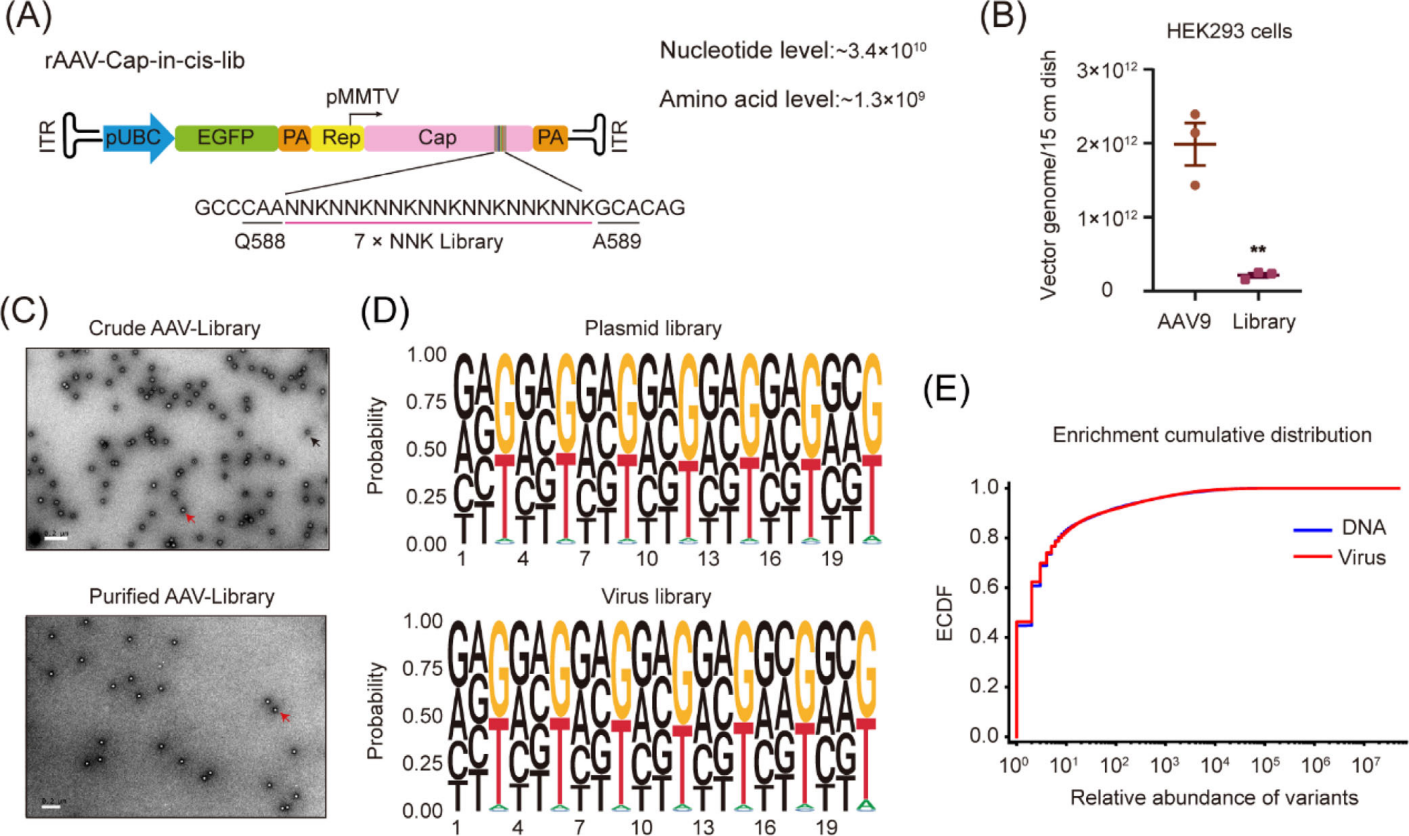
Construction of AAV9 capsid library. (A) Map of self‐packaging capsid library construction. (B) Comparison of rAAV production between wild‐type AAV9 and self‐packaging capsid library. Data are presented as mean ± SEM (*n* = 3), *p < 0.05; **p < 0.01 (unpaired *t*‐test). (C) Analysis of AAV virus library via transmission electron microscopy (TEM) before and after purification. Red and black arrows denote full and empty AAV particles respectively. Scale bar, 0.2 μm. (D) Base distribution of plasmid DNA library and virus library by next‐generation sequencing (NGS). (E) Empirical cumulative distribution frequency (ECDF) of plasmid DNA library and virus library.

As mentioned above, we selected Q588 and A589 (the VP1 position) in native AAV9 and inserted a synthetic sequence pool of seven random, unnatural amino acids (Figure 2A). Our synthetic pool was formed by random DNA oligo, which meant 21 oligo (corresponding to codons of seven amino acids) were replaced by nucleotides randomly supplemented with equal chance of incorporation among the four bases of A, T, C and G. The advantage is that the synthesized oligo library has a large theoretical capacity, which contains approximately 3.4 × 10^10^ and 1.3 × 10^9^ variants at nucleotide level and amino acid level, respectively. Concurrently, the synthesis time takes only 3 working days, the same as rapid primer synthesis.

The capsid library construct was flanked by inverted terminal repeats (ITRs), thereby eliciting self‐packaging of the cap gene so that each capsid variant packaged its own coding sequence as a transgene. AAV variant library was generated from HEK293 cells transfected by capsid library plasmid and a modified AAV2/9 Rep‐Cap helper plasmid (Figures 2A and S1A), indicating that the recombinant AAV maintained the ability of virus particle formation after the insertion sequence. Viral titer assay showed that the vector containing the synthetic sequence could yield AAV particles in a slightly lower yield than that of the wild type owing to plasmid transfection limit (Figure 2B). Electron microscopy analysis showed that the recombinant AAV had the same particle structure as the wild type, and there was significant difference of full/empty ratio between the virus before and after purification (Figure 2C). The purified viral particles were digested with protease, and then the internal packaging DNA sequences were analysed by next generation sequencing. Both the DNA library and AAV library had a uniform distribution at nucleotide level and amino acid level (Figures 2D and S1B). Empirical cumulative distribution frequency (ECDF) showed that the process of viral packaging did not amplify the difference between the variants (Figure 2E).

### 3.2 Generating adult chimeric mice with human hepatocytes for in vivo virus screening

**FIGURE 3:**
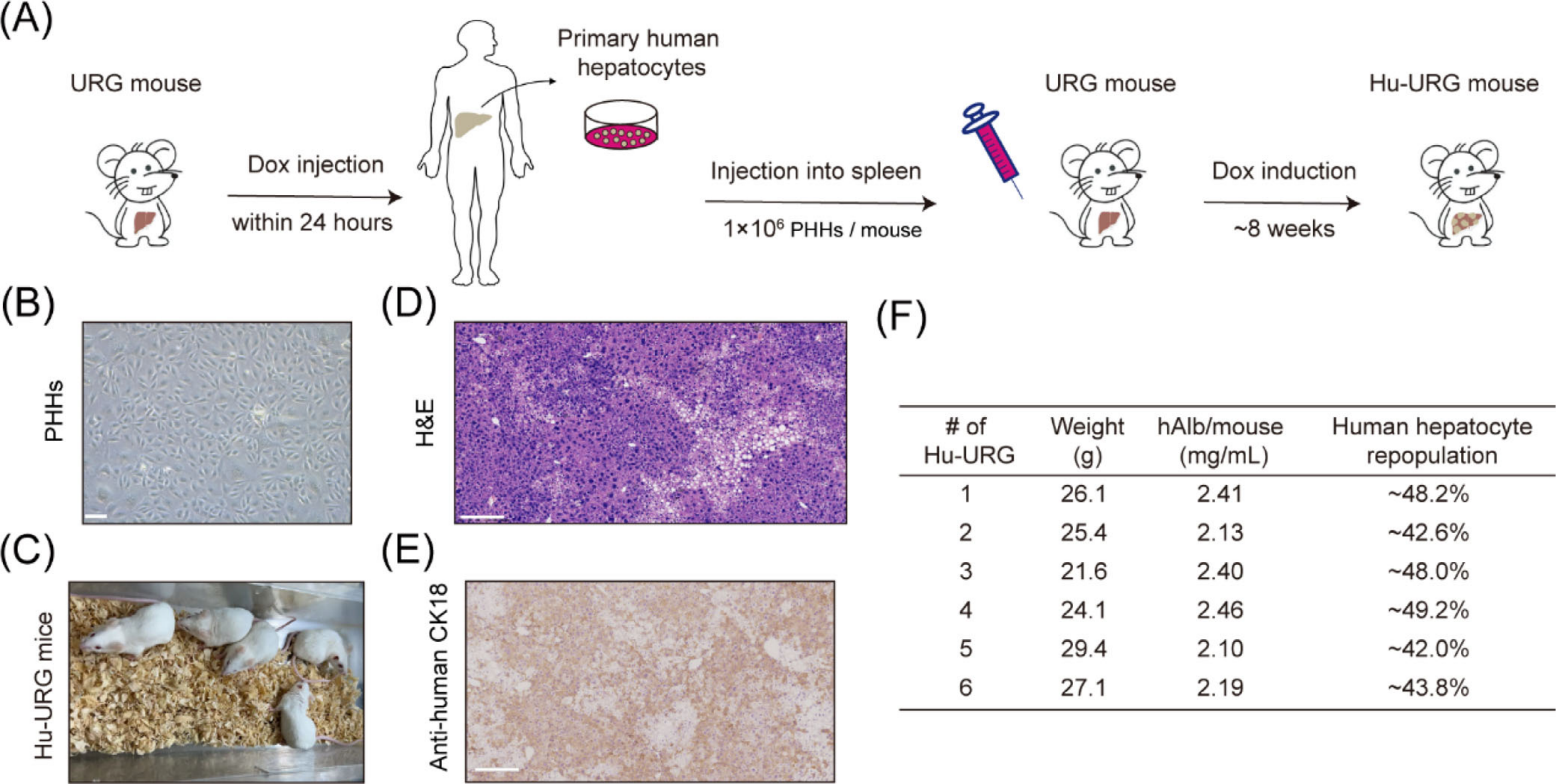
Hu‐URG liver‐humanized mice in AAV variant selection. (A) Schematic diagram of liver‐humanized mouse. URG mouse, Tet‐uPA^Tg^ Rag2^null^ Il2rg^null^ mouse. Hu‐URG mouse, humanized URG mouse. PHHs, primary human hepatocytes. (B) Primary human hepatocytes isolated from the donor before transplantation. Scale bar, 100 μm. (C) Humanized URG mice after transplantation. (D) Hematoxylin–eosin (H&E) staining shows the formation of human‐mouse chimeric liver, and the lightly stained part shows reconstitution of human hepatocytes. Scale bar, 200 μm. (E) Representative images of immunohistochemically stained chimeric liver shows the expression of human‐specific marker (CK18) in liver‐humanized mice. Scale bar, 200 μm. (F) Human serum albumin levels and percent transduction efficiency for individual humanized mice.

In this study, we used a relatively sophisticated mouse model as the subject of in vivo AAV screening. These mice were generated by mating NRG mice possessing a genetic defect (Rag2/Il2rg) conferring immunodeficiency, and Alb‐rtTA/TRE‐uPA mice capable of producing endogenous liver damage in the presence of doxycycline.^37^ In order to establish human hepatocytes contained within the context of an intact liver in an animal model, we injected the Tet‐uPA^Tg^ Rag2^null^ Il2rg^null^ (URG) mice with human hepatocytes (Figure 3A). The URG mice were raised under sterile conditions until old enough for transplantation (8‐weeks old). Approximately 1.0 × 10^6^ PHHs isolated from the donor were injected into the inferior splenic pole of mice, the endogenous hepatocytes of which were damaged by doxycycline 24 hours before transplantation (Figure 3A,B). Subsequently, doxycycline was added to drinking water to induce continuous liver injury. All URG mice transplanted with human hepatocytes survived as humanized URG (Hu‐URG) mice (Figure 3C). Eight weeks after hepatocyte transplantation, human serum albumin levels in peripheral blood of Hu‐URG mice was measured by ELISA, indicating that the proportion of human liver cells in these mice ranged from 42.0% to 49.2% (Figure 3F). Subsequent sectioning and immunohistochemical (IHC) results also confirmed the presence of extensive human liver cells detected in the livers of these mice (Figure 3D,E).

### 3.3 Yielding AAV9 variants with enhanced hepatocyte transduction

**FIGURE 4:**
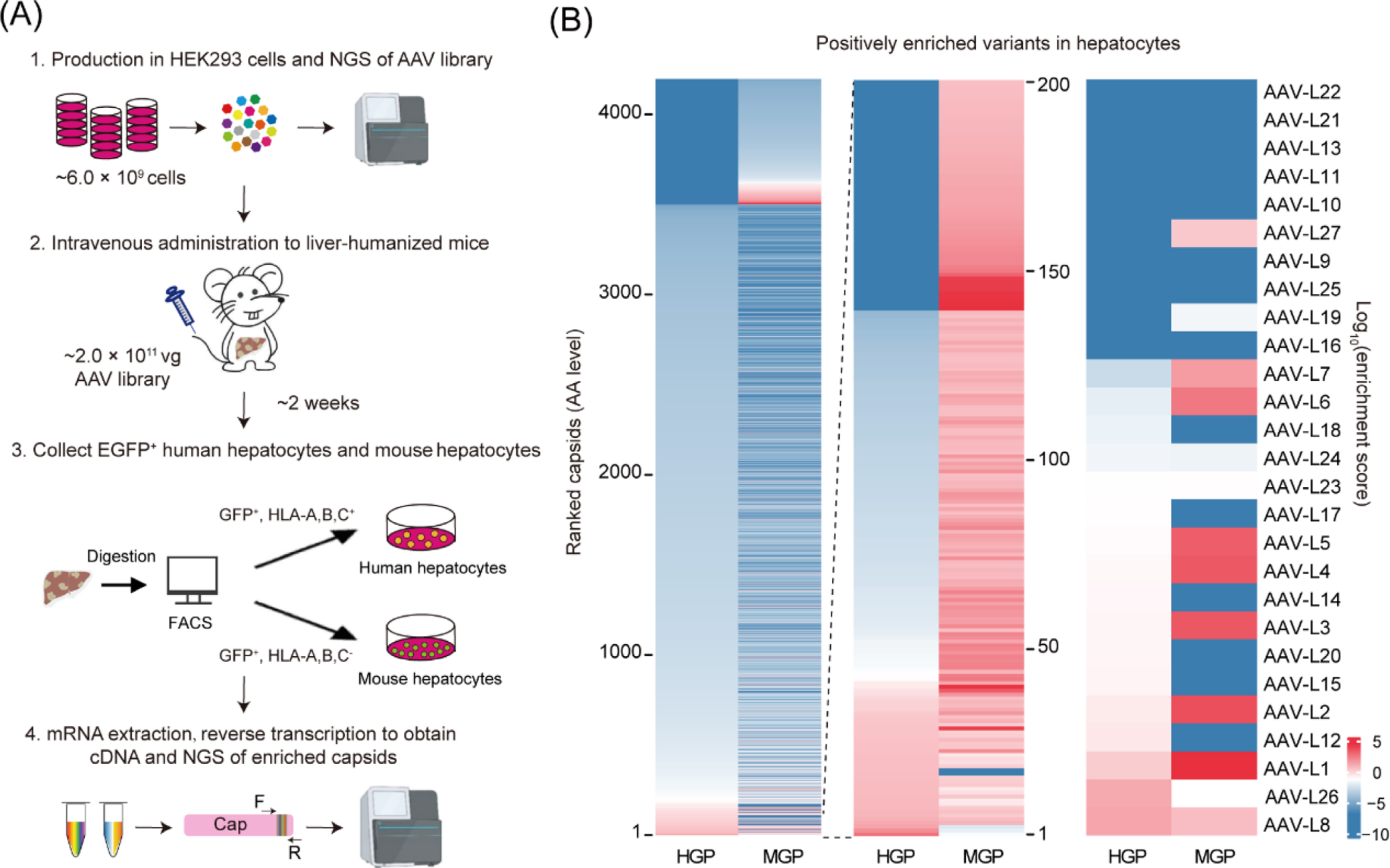
Directed evolution of AAV9 in liver‐humanized mice yields variants for enhanced human hepatocyte transduction. (A) Schematic of AAV capsid library evolution in liver‐humanized mice following intravenous administration by tail. FACS, Fluorescence‐activated cell sorting. NGS, next‐generation sequencing. (B) Heat map of relative enrichment for variants enriched in the human and mouse hepatocytes (left). Zoom‐in of the most enriched variants (middle), and of the variants that are characterized in the current study (right) are shown. HGP, human EGFP positive hepatocytes; MGP, mouse EGFP positive hepatocytes.

As mentioned above, AAV capsid libraries were produced in HEK293 cells. To improve the number of each packable variant, we repeated the experiment more than 10 times, yielding a total of 4.8 × 10^11^ virus particles. These AAV9 variant virus particles with DNA sequence information inserted with specific VP3 variants were sufficient to infect two mice. We described the screening procedures for a full round (Figure 4A), wherein Hu‐URG mice received 2.0 × 10^11^ vg/animal AAV library via intravenous injection, and 2 weeks later, the liver tissue was isolated and digested into a single‐cell suspension for Fluorescence‐Activated Cell Sorting (FACS). We then extracted RNA from sorted EGFP‐positive hepatocytes and recovered capsid sequences through PCR and next‐generation sequencing (NGS).

Upon deep sequencing, we observed ~4 × 10^4^ unique nucleotide variants in human hepatocytes samples, and each variant was represented with an enrichment score that reflects the change in relative abundance between the liver and the starting virus library. Then, we synthesized ~8405 variant sequences with the highest enrichment ranking as the library for the second round of screening. More than 7800 variants (~94%) were sequenced in the plasmid library, and approximately 7552 variants (~90%) were found after viral production, indicating that most variants had acceptable production efficiency (Figure S2A). Compared with the first round of library, increased heterogeneity was observed in the second one. The DNA library is more evenly distributed, indicating enrichment of a subset of variants during viral production (Figure S2B). This may be caused by cell transfection preference during packaging, or that different sequences have a certain bias for virus packaging ability. After the second round of selection, we observed more than 4000 unique nucleotide variants in the sorted EGFP‐positive cells, ~83% of which were identified in the sequenced portion of human hepatocytes (Figure 4B). To identify variants that transduce hepatocytes more efficiently, we chose 27 variants with good reproducibility and production from the top 5% ranked by average enrichment score in human and mouse hepatocytes for validation (Figure 4B).

### 3.4 AAV‐L8 enables efficient transduction of human hepatocytes

**FIGURE 5:**
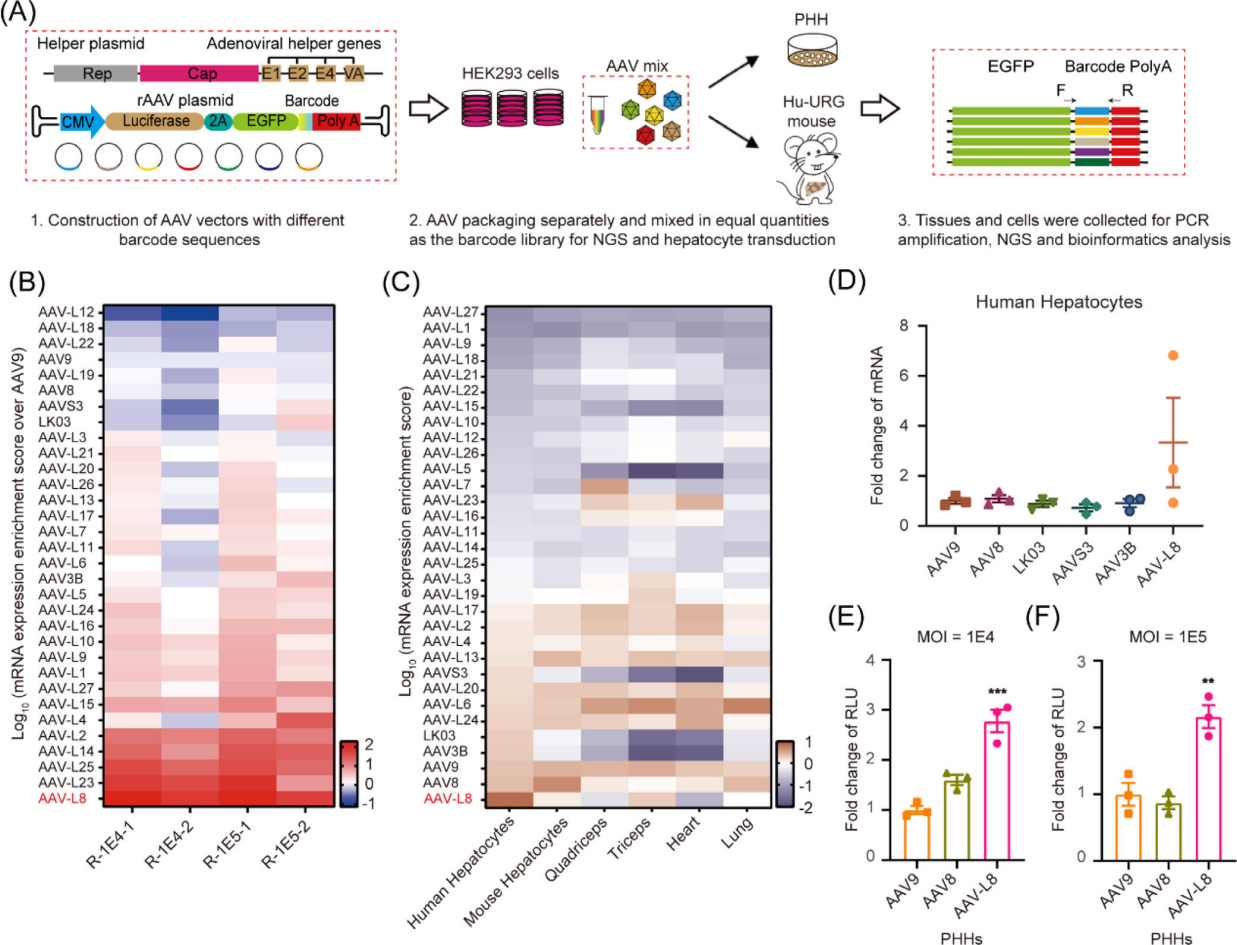
In vivo and in vitro characterization of AAV vectors yields AAV‐L8. (A) Schematic of the barcoded EGFP transgene and strategy for assessing the performance of top enriched variants in liver‐humanized mice and primary human hepatocytes. PHH, primary human hepatocytes. NGS, next‐generation sequencing. (B) The heat map shows fold change of mRNA expression enrichment score over AAV9 in primary human hepatocytes (PHHs). R, RNA sample. 1E4 and 1E5 represent values of multiplicity of infection (MOI). (C) Heat map shows distinct tropism of variants across organs at mRNA expression level. (D) Fold difference in within‐individual EGFP mRNA expression from different variants normalized to AAV9 in human hepatocytes of three liver humanized mice. (E, F) Transduction evaluation of AAV‐L8 in PHHs at MOI of 1E4 (E) and 1E5 (F). RLU, relative luminescence units. Data are presented as mean ± SEM (*n* = 3), *p < 0.05; **p < 0.01; ***p < 0.001 (one‐way ANOVA using Dunnett's multiple comparison test).

Choosing the best rAAV serotype for optimum human hepatic delivery has grown increasingly complex and controversial in recent years due to differences in experimental setup, data interpretation and reproducibility.^38^, ^39^, ^40^, ^41^ Thus, apart from AAV9, four serotypes AAV8, LK03, AAVS3 and AAV3B with strong transduction ability in liver cells were selected as controls. To evaluate the transduction ability of AAV9 variants both in vitro and in vivo while minimizing the individual differences between animals, we adopted a new method (Figure 5A). Each variant was used to package a CMV‐luciferase‐EGFP cassette carrying a specific barcode sequence. The 32 viruses were packaged and mixed in nearly equal proportions to create a virus library with different barcode sequences, which was utilized for both in vitro and in vivo infection experiments. The enrichment score of each variant was calculated based on the relative ratio of barcode sequences in the cell or tissue samples compared to those in the mixed virus.

For in vitro validation, primary human hepatocytes (PHHs) were infected at two MOI values, 1E4 and 1E5. The hepatocyte transduction of each variant was evaluated at the DNA and mRNA expression levels (Figures 5B and S3). The mRNA‐level enrichment scores revealed that approximately 24 variants exhibited higher transduction efficiency in PHH compared to AAV9, with AAV‐L8 having the highest enrichment score at both MOI values (Figure 5B).

The results of in vivo validation are more precise than those obtained through in vitro validation. Thus, three Hu‐URG mice were intravenously injected with a dose of 2 × 10^12^ vg per mouse through the tail vein. Two weeks later, EGFP‐positive liver cells were sorted by FACS. Meanwhile, the heart, lung, kidney, muscle and other tissues were collected from mice for mRNA level analysis of variant enrichment (Figure 5C). Although most capsid variants outperformed AAV9 in vitro (Figures 5B and S3), only AAV‐L8 showed higher transduction efficiency than AAV9 in human hepatocytes of humanized mice (Figure 5C). The performance of AAV‐L8 in human hepatocytes was exceptional, exhibiting a 3.3‐fold higher transduction at the mRNA level compared to the five control serotypes (Figure 5D).

The transgene barcode pool validation strategy is limited by RNA level. To evaluate the transduction efficiency at a more comprehensible protein level, AAV‐L8 was further validated by transducing PHHs and two human liver carcinoma cell lines with individual AAV vectors carrying the CMV‐luciferase‐EGFP cassette. We compared the transduction ability of AAV‐L8 with parental AAV9 and wild‐type AAV8 by analysing the luciferase activity of the infected cells. Reaffirming the findings in liver‐humanized mice, AAV‐L8 demonstrated higher transduction efficiency in PHHs. At an MOI of 1E4, the luciferase relative luminescence units (RLU) of AAV‐L8 were approximately 2.8‐fold higher than that of AAV9 (Figure 5E). At an MOI of 1E5, the RLU values for AAV‐L8 were 2.2‐fold and 2.5‐fold higher than those for AAV9 and AAV8, respectively (Figure 5F). Moreover, AAV‐L8 exhibited the highest transduction efficiency in Huh7 and HepG2 cell lines. The luciferase RLU of AAV‐L8 exhibited an approximate 4‐fold increase compared to AAV9 in Huh7 cell line at both MOIs (Figure S4A,B). In HepG2 cell line, the luciferase RLU of AAV‐L8 were approximately 2.8‐fold and 2‐fold higher than AAV9 at the MOI of 1E4 and 1E5, respectively (Figure S4C,D). Thus, the observed discrepancy in transduction efficiencies between AAV‐L8 and wild‐type serotypes in three human liver cell lines substantiates the augmented transduction capacity of AAV‐L8 in human hepatocytes.

### 3.5 AAV‐L8 yields human hepatocyte‐specific transgene expression in mice

**FIGURE 6:**
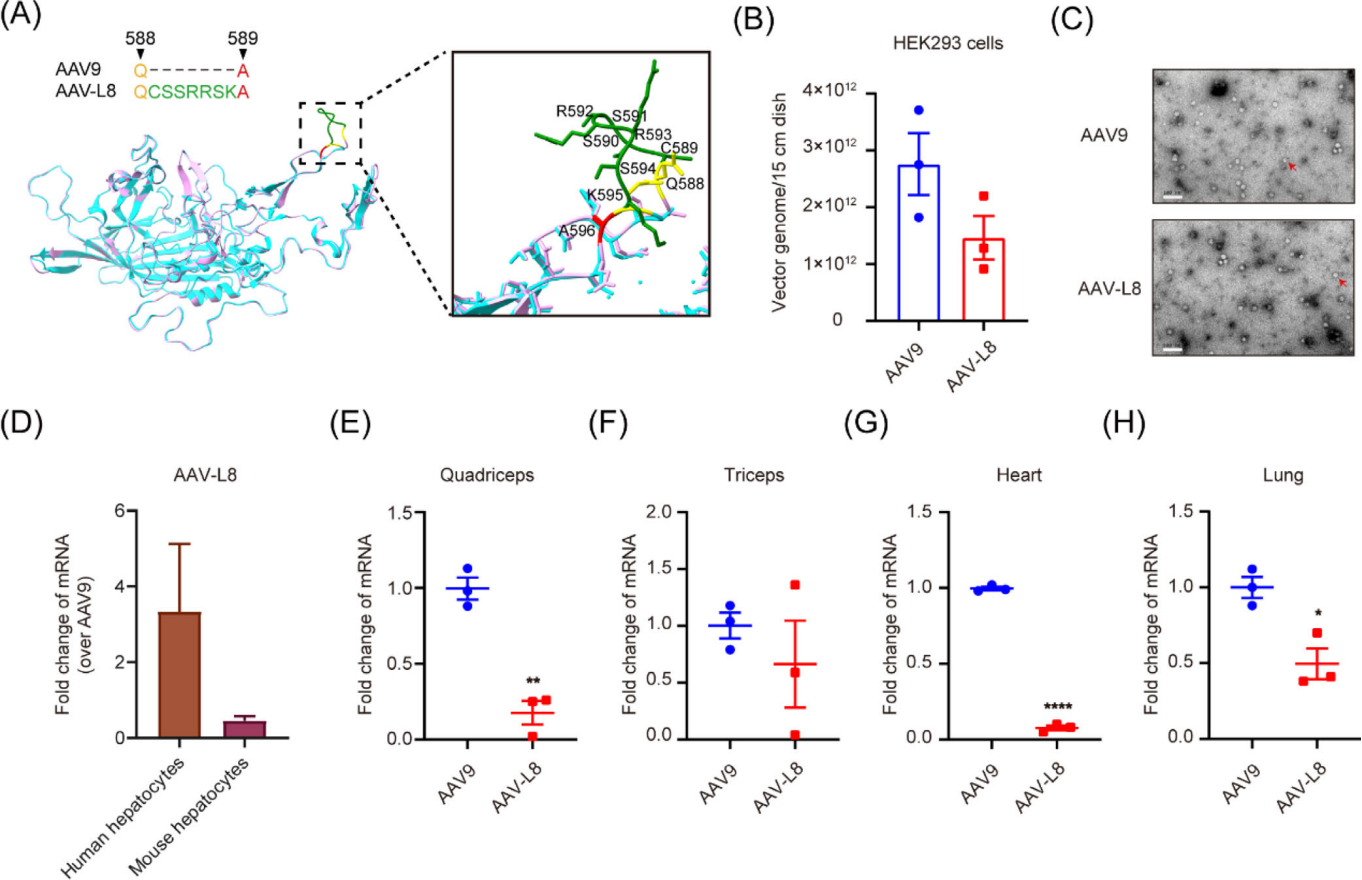
AAV‐L8 shows lower expression in peripheral tissues of mouse. (A) Predicted structure comparison between AAV9 and AAV‐L8 capsid. The VR‐VIII surface loops are magnified in the black rectangle frame (right). Computational modelling of the VR‐VIII loop of AAV‐L8 in purple was performed on the ProMod3‐powered SWISS‐MODEL server. AAV9 in cyan was used as a template for homology modelling (PDB: 3UX1) and all structures were visualized in ChimeraX. (B) Evaluation of the packaging efficiency between two serotypes. (C) Analysis of AAV9 and AAV‐L8 via transmission electron microscopy (TEM). Red arrows denote full AAV particles of AAV9 and AAV‐L8. Scale bar, 100 nm. (D) Fold change of mRNA expression over AAV9 in human and mouse hepatocytes. (E, H) Fold change of mRNA expression over AAV9 in quadriceps (E), triceps (F), heart (G) and lung (H). Data are presented as mean ± SEM (*n* = 3), *p < 0.05; **p < 0.01; ***p < 0.001; ****p < 0.0001 (unpaired *t*‐test).

As alluded to earlier, peptide insertion at VR‐VIII did not change the capsid assembly.^33^ The variant AAV‐L8 is predicted to have similar but distinct secondary structures in the VR‐VIII loop region compared to the parental AAV9 (Figure 6A). Evaluation of production efficiency in HEK293 cells showed a marginally decreased of AAV‐L8, but with no significance (Figure 6B). Transmission electron microscopy (TEM) analysis showed no significant difference of full/empty ratio between AAV9 and AAV‐L8 (Figure 6C).

Furthermore, AAV‐L8, which is highly effective in human hepatocytes, transduces mouse hepatocytes approximately 2‐times less efficiently than AAV9 (Figure 6D). Decreased levels of AAV‐L8 were detected in mouse hepatocytes upon analysis of transgene mRNA expression (Figure S5A) and vector genome delivery (Figure S5B). As mentioned above, the insertion site is located at the protrusions of the capsid's threefold symmetry axis structure, which facilitates the interactions between inserted peptides and membrane molecules on target cells. Based on the competition among cell membrane surface receptors, the peptide insertion may drive the gene expression changes in other organs. Compared with AAV9, AAV‐L8 showed significant decreased transgene expression in the quadriceps (Figure 6E). The same trend has also been detected in tricep (Figure 6F), heart (Figure 6G) and lung tissues (Figure 6H). Taken together, these data demonstrate that through systemic administration, AAV‐L8 can improve the human hepatocyte transduction of AAV9 while reducing its transduction of other tissues, which is conducive to improving the safety of AAV9 in clinical applications. We additionally provide a list of the 50 most highly enriched capsid variants in mouse hepatocytes (Table S3). Further investigation and characterization of these variants may identify additional candidates for liver gene therapy.

## 4 DISCUSSION

AAV9, discovered in the human liver tissue,^21^ is commonly used in preclinical and clinical trials of the CNS, muscles and liver owing to its broad tropism. Directed evolution of AAV9 through random peptide insertion can effectively guide capsid engineering. Using this strategy, previous studies have identified MyoAAV mutants with potent muscle transduction and AAV‐MG mutants with high microglial infection efficiency.^42^, ^43^ However, there have been no reports on improving the liver transduction of AAV9 using peptide insertion. Based on the successful engineering cases mentioned above, it is possible to yield variants with improved liver transduction through directed evolution of AAV9. By optimizing the screening approach, we characterized novel variants with enhanced liver transduction and specificity.

Concerned that the possible sequence bias during viral production and recovery would propagate across selection rounds, we designed an unbiased library based on the first round output (synthetic pool library) via oligonucleotide pools. Comparatively, the synthetic pool DNA library is more evenly distributed, minimizing bias amplification across selection rounds. Consequently, the evenly distributed feature of synthetic pool avoids obscuring the true enrichment of variants during in vivo selection and reduces the number of screening rounds, thereby shortening the overall screening cycle.

Although the liver anatomy is similar between mouse and human,^44^ there are differences in transcriptome, enzyme activities and non‐conserved genes,^45^, ^46^ making it challenging to evaluate functions of human‐applicable AAV using mouse models. Even with in vivo transduction measurements, utilization of different species without human xenografts inadequately replicates transduction outcomes observed in humans.^18^, ^47^ Current evidence suggests that assessing functional transduction in a xenograft liver model transplanted with primary human hepatocytes provides the closest representation to available patient data.^39^, ^40^ In this study, URG mice were transplanted with PHHs to serve as our screening and validation animal models, screening out AAV variants with enhanced human hepatocyte transduction (Figure 3A).

Previous studies have showed that the accumulation of virus particles is not necessarily correlated with transgenic expression levels.^48^ AAV transduction is a multi‐step process including binding to receptors on the cell surface, intracellular trafficking, endosomal escape, nuclear entry, second strand DNA synthesis and transgene expression, and any of these steps can limit vector potency.^49^, ^50^ For example, although AAV8 is highly hepatophilic, its ability to express foreign genes after entering hepatocytes is weak.^51^ However, the majority of in vivo capsid‐directed evolution strategies select capsid variants based on their presence in the target tissue of the vector genome DNA, rather than the transgene mRNA.^19^ The capsid‐directed evolution screening and validation strategies in our study were founded on the level of transgene mRNA expression necessary for all stages of rAAV transduction, ensuring efficient transgene expression (Figures 4A and 5A).

In our study, a multiplex barcode recombinant AAV variant‐tracing strategy and currently used tools for human hepatocyte studies, including immortalized hepatocyte cell lines, PHHs and liver‐humanized mice, were applied to transduction efficiency validation. The identification of variants with enhanced hepatocyte transduction and specific tropism only required two rounds of selection (Figures 1B and 4A), owing to oligonucleotide pool, optimal animal model, quantification of transgene mRNA expression and in vitro‐in vivo validation strategies, which reduce both cost and time. The optimized screening approach can also be applied to the engineering of AAV, VLP and other delivery vectors.

In summary, we characterized novel variants derived from AAV9 using the optimized screening approach, which suggest that rAAV‐based therapies with new variants may achieve therapeutic efficacy at a lower dose, minimizing both safety concerns and vector manufacturing challenges. Thus, our results, together with those of previous studies,^39^, ^52^ bridge the gap in our knowledge of AAV capsid engineering in animal models and clinical trials, contributing to gene and cell therapy, cell fate determination, reversing senility and regenerative medicine research.

## AUTHOR CONTRIBUTIONS

Wei Li conceived the idea; Moyu Dai and Kai Xu designed and performed the experiments; Ning Yang performed bioinformatics analysis; Jingwen Zhang and Xueke Li helped with AAV production and animal experiments; Wei Li, Ying Zhang, Moyu Dai and Kai Xu performed manuscript writing, review and editing. All authors read and approved the final manuscript.

## FUNDING INFORMATION

This study was supported by grants from the National Key Research and Development Program (2019YFA0110800 to W.L., 2019YFA0903800 and 2020YFA0707900), the CAS Project for Young Scientists in Basic Research (YSBR‐012 to W.L.) and the National Postdoctoral Program for Innovative Talents (BX20200333 to K.X.).

## CONFLICT OF INTEREST STATEMENT

The authors declare no conflict of interest.

## Supporting information


**Data S1.** Supporting Information

## Data Availability

The data used to support the findings of this study are available from the authors upon reasonable request.

## References

[1] Verma IM , Somia N . Gene therapy – promises, problems and prospects. Nature. 1997;389:239‐242. 9305836 10.1038/38410

[2] Hoy SM . Onasemnogene abeparvovec: first global approval. Drugs. 2019;79:1255‐1262. 31270752 10.1007/s40265-019-01162-5

[3] Mendell JR , Al‐Zaidy SA , Rodino‐Klapac LR , et al. Current clinical applications of in vivo gene therapy with AAVs. Mol Ther. 2021;29:464‐488. 33309881 10.1016/j.ymthe.2020.12.007 PMC7854298

[4] Frangoul H , Altshuler D , Cappellini MD , et al. CRISPR‐Cas9 gene editing for sickle cell disease and beta‐thalassemia. N Engl J Med. 2021;384:252‐260. 33283989 10.1056/NEJMoa2031054

[5] Liu XS , Wu H , Ji X , et al. Editing DNA methylation in the mammalian genome. Cell. 2016;167:233‐247.e17. 27662091 10.1016/j.cell.2016.08.056 PMC5062609

[6] Montana CL , Kolesnikov AV , Shen SQ , Myers CA , Kefalov VJ , Corbo JC . Reprogramming of adult rod photoreceptors prevents retinal degeneration. Proc Natl Acad Sci U S A. 2013;110:1732‐1737. 23319618 10.1073/pnas.1214387110 PMC3562787

[7] Yao K , Qiu S , Wang YV , et al. Restoration of vision after de novo genesis of rod photoreceptors in mammalian retinas. Nature. 2018;560:484‐488. 30111842 10.1038/s41586-018-0425-3 PMC6107416

[8] Yu W , Mookherjee S , Chaitankar V , et al. Nrl knockdown by AAV‐delivered CRISPR/Cas9 prevents retinal degeneration in mice. Nat Commun. 2017;8:14716. 28291770 10.1038/ncomms14716 PMC5355895

[9] Zhu J , Ming C , Fu X , et al. Gene and mutation independent therapy via CRISPR‐Cas9 mediated cellular reprogramming in rod photoreceptors. Cell Res. 2017;27:830‐833. 28429769 10.1038/cr.2017.57 PMC5518875

[10] Browder KC , Reddy P , Yamamoto M , et al. In vivo partial reprogramming alters age‐associated molecular changes during physiological aging in mice. Nat Aging. 2022;2:243‐253. 37118377 10.1038/s43587-022-00183-2

[11] Kurita M , Araoka T , Hishida T , et al. In vivo reprogramming of wound‐resident cells generates skin epithelial tissue. Nature. 2018;561:243‐247. 30185909 10.1038/s41586-018-0477-4 PMC9651909

[12] Lu Y , Brommer B , Tian X , et al. Reprogramming to recover youthful epigenetic information and restore vision. Nature. 2020;588:124‐129. 33268865 10.1038/s41586-020-2975-4 PMC7752134

[13] Rodriguez‐Matellan A , Alcazar N , Hernandez F , Serrano M , Avila J . In vivo reprogramming ameliorates aging features in dentate gyrus cells and improves memory in mice. Stem Cell Reports. 2020;15:1056‐1066. 33096049 10.1016/j.stemcr.2020.09.010 PMC7663782

[14] Ocampo A , Reddy P , Martinez‐Redondo P , et al. In vivo amelioration of age‐associated hallmarks by partial reprogramming. Cell. 2016;167:1719‐1733.e12. 27984723 10.1016/j.cell.2016.11.052 PMC5679279

[15] Ribeiro R , Macedo JC , Costa M , et al. In vivo cyclic induction of the FOXM1 transcription factor delays natural and progeroid aging phenotypes and extends healthspan. Nat Aging. 2022;2:397‐411. 37118067 10.1038/s43587-022-00209-9

[16] Yang JH , Hayano M , Griffin PT , et al. Loss of epigenetic information as a cause of mammalian aging. Cell. 2023;186:305‐326.e327. 36638792 10.1016/j.cell.2022.12.027 PMC10166133

[17] Manno CS , Pierce GF , Arruda VR , et al. Successful transduction of liver in hemophilia by AAV‐factor IX and limitations imposed by the host immune response. Nat Med. 2006;12:342‐347. 16474400 10.1038/nm1358

[18] Nathwani AC , Tuddenham EGD , Rangarajan S , et al. Adenovirus‐associated virus vector‐mediated gene transfer in hemophilia B. N Engl J Med. 2011;365:2357‐2365. 22149959 10.1056/NEJMoa1108046 PMC3265081

[19] Li C , Samulski RJ . Engineering adeno‐associated virus vectors for gene therapy. Nat Rev Genet. 2020;21:255‐272. 32042148 10.1038/s41576-019-0205-4

[20] Wang D , Tai PWL , Gao G . Adeno‐associated virus vector as a platform for gene therapy delivery. Nat Rev Drug Discov. 2019;18:358‐378. 30710128 10.1038/s41573-019-0012-9 PMC6927556

[21] Gao G , Vandenberghe LH , Alvira MR , et al. Clades of adeno‐associated viruses are widely disseminated in human tissues. J Virol. 2004;78:6381‐6388. 15163731 10.1128/JVI.78.12.6381-6388.2004 PMC416542

[22] Miesbach W , Meijer K , Coppens M , et al. Gene therapy with adeno‐associated virus vector 5‐human factor IX in adults with hemophilia B. Blood. 2018;131:1022‐1031. 29246900 10.1182/blood-2017-09-804419 PMC5833265

[23] Pasi KJ , Rangarajan S , Mitchell N , et al. Multiyear follow‐up of AAV5‐hFVIII‐SQ gene therapy for hemophilia a. N Engl J Med. 2020;382:29‐40. 31893514 10.1056/NEJMoa1908490

[24] Wilson JM , Flotte TR . Moving forward after two deaths in a gene therapy trial of myotubular myopathy. Hum Gene Ther. 2020;31:695‐696. 32605399 10.1089/hum.2020.182

[25] Kuzmin DA , Shutova MV , Johnston NR , et al. The clinical landscape for AAV gene therapies. Nat Rev Drug Discov. 2021;20:173‐174. 33495615 10.1038/d41573-021-00017-7

[26] Rodriguez‐Marquez E , Meumann N , Buning H . Adeno‐associated virus (AAV) capsid engineering in liver‐directed gene therapy. Expert Opin Biol Ther. 2021;21:749‐766. 33331201 10.1080/14712598.2021.1865303

[27] Chiorini JA , Kim F , Yang L , Kotin RM . Cloning and characterization of adeno‐associated virus type 5. J Virol. 1999;73:1309‐1319. 9882336 10.1128/jvi.73.2.1309-1319.1999 PMC103955

[28] Farris KD , Pintel DJ . Improved splicing of adeno‐associated viral (AAV) capsid protein‐supplying pre‐mRNAs leads to increased recombinant AAV vector production. Hum Gene Ther. 2008;19:1421‐1427. 18785816 10.1089/hum.2008.118 PMC2940631

[29] Deverman BE , Pravdo PL , Simpson BP , et al. Cre‐dependent selection yields AAV variants for widespread gene transfer to the adult brain. Nat Biotechnol. 2016;34:204‐209. 26829320 10.1038/nbt.3440 PMC5088052

[30] Dandri M , Burda MR , Török E , et al. Repopulation of mouse liver with human hepatocytes and in vivo infection with hepatitis B virus. Hepatology. 2001;33:981‐988. 11283864 10.1053/jhep.2001.23314

[31] Tateno C , Kawase Y , Tobita Y , et al. Generation of novel chimeric mice with humanized livers by using Hemizygous cDNA‐uPA/SCID mice. PLoS One. 2015;10:e0142145. 26536627 10.1371/journal.pone.0142145 PMC4633119

[32] Ravindra Kumar S , Miles TF , Chen X , et al. Multiplexed Cre‐dependent selection yields systemic AAVs for targeting distinct brain cell types. Nat Methods. 2020;17:541‐550. 32313222 10.1038/s41592-020-0799-7 PMC7219404

[33] DiMattia MA , Nam HJ , van Vliet K , et al. Structural insight into the unique properties of adeno‐associated virus serotype 9. J Virol. 2012;86:6947‐6958. 22496238 10.1128/JVI.07232-11 PMC3393551

[34] Rose JA , Maizel JV Jr , Inman JK , Shatkin AJ . Structural proteins of adenovirus‐associated viruses. J Virol. 1971;8:766‐770. 5132697 10.1128/jvi.8.5.766-770.1971 PMC376258

[35] Opie SR , Warrington KH Jr , Agbandje‐McKenna M , Zolotukhin S , Muzyczka N . Identification of amino acid residues in the capsid proteins of adeno‐associated virus type 2 that contribute to heparan sulfate proteoglycan binding. J Virol. 2003;77:6995‐7006. 12768018 10.1128/JVI.77.12.6995-7006.2003 PMC156206

[36] Govindasamy L , DiMattia MA , Gurda BL , et al. Structural insights into adeno‐associated virus serotype 5. J Virol. 2013;87:11187‐11199. 23926356 10.1128/JVI.00867-13 PMC3807309

[37] Song X , Guo Y , Duo S , et al. A mouse model of inducible liver injury caused by tet‐on regulated urokinase for studies of hepatocyte transplantation. Am J Pathol. 2009;175:1975‐1983. 19808649 10.2353/ajpath.2009.090349 PMC2774061

[38] Kay MA . Selecting the best AAV capsid for human studies. Mol Ther. 2015;23:1800‐1801. 26689120 10.1038/mt.2015.206 PMC4700120

[39] Lisowski L , Dane AP , Chu K , et al. Selection and evaluation of clinically relevant AAV variants in a xenograft liver model. Nature. 2014;506:382‐386. 24390344 10.1038/nature12875 PMC3939040

[40] Vercauteren K , Hoffman BE , Zolotukhin I , et al. Superior in vivo transduction of human hepatocytes using engineered AAV3 capsid. Mol Ther. 2016;24:1042‐1049. 27019999 10.1038/mt.2016.61 PMC4923326

[41] Wang L , Bell P , Somanathan S , et al. Comparative study of liver gene transfer with AAV vectors based on natural and engineered AAV capsids. Mol Ther. 2015;23:1877‐1887. 26412589 10.1038/mt.2015.179 PMC4700115

[42] Lin R , Zhou Y , Yan T , et al. Directed evolution of adeno‐associated virus for efficient gene delivery to microglia. Nat Methods. 2022;19:976‐985. 35879607 10.1038/s41592-022-01547-7

[43] Tabebordbar M , Lagerborg KA , Stanton A , et al. Directed evolution of a family of AAV capsid variants enabling potent muscle‐directed gene delivery across species. Cell. 2021;184:4919‐4938.e22. 34506722 10.1016/j.cell.2021.08.028 PMC9344975

[44] Kruepunga N , Hakvoort TBM , Hikspoors J , Kohler SE , Lamers WH . Anatomy of rodent and human livers: what are the differences? Biochim Biophys Acta Mol Basis Dis. 2019;1865:869‐878. 29842921 10.1016/j.bbadis.2018.05.019

[45] Martignoni M , Groothuis GM , de Kanter R . Species differences between mouse, rat, dog, monkey and human CYP‐mediated drug metabolism, inhibition and induction. Expert Opin Drug Metab Toxicol. 2006;2:875‐894. 17125407 10.1517/17425255.2.6.875

[46] Yu Y , Ping J , Chen H , et al. A comparative analysis of liver transcriptome suggests divergent liver function among human, mouse and rat. Genomics. 2010;96:281‐289. 20800674 10.1016/j.ygeno.2010.08.003

[47] Nathwani AC , Reiss UM , Tuddenham EGD , et al. Long‐term safety and efficacy of factor IX gene therapy in hemophilia B. N Engl J Med. 2014;371:1994‐2004. 25409372 10.1056/NEJMoa1407309 PMC4278802

[48] Westhaus A , Cabanes‐Creus M , Rybicki A , et al. High‐throughput in vitro, ex vivo, and in vivo screen of adeno‐associated virus vectors based on physical and functional transduction. Hum Gene Ther. 2020;31:575‐589. 32000541 10.1089/hum.2019.264 PMC7232709

[49] Berry GE , Asokan A . Cellular transduction mechanisms of adeno‐associated viral vectors. Curr Opin Virol. 2016;21:54‐60. 27544821 10.1016/j.coviro.2016.08.001 PMC5138113

[50] Ding W , Zhang L , Yan Z , Engelhardt JF . Intracellular trafficking of adeno‐associated viral vectors. Gene Ther. 2005;12:873‐880. 15829993 10.1038/sj.gt.3302527

[51] Konkle BA , Walsh CE , Escobar MA , et al. BAX 335 hemophilia B gene therapy clinical trial results: potential impact of CpG sequences on gene expression. Blood. 2021;137:763‐774. 33067633 10.1182/blood.2019004625 PMC7885820

[52] Grimm D , Lee JS , Wang L , et al. In vitro and in vivo gene therapy vector evolution via multispecies interbreeding and retargeting of adeno‐associated viruses. J Virol. 2008;82:5887‐5911. 18400866 10.1128/JVI.00254-08 PMC2395137

