# Supplementary material for: Discovering human cell‐compatible gene therapy virus variants via optimized screening in mouse models
Source: Cell Prolif. 2023 Oct 20;57(3):e13565. doi: 10.1111/cpr.13565 (PMC10905335; doi:10.1111/cpr.13565)
Supplement: Supplementary file 1 — Data S1. Supporting Information [file CPR-57-e13565-s001.docx]

**Supplementary information**

**MATERIALS AND METHODS**

**AAV plasmid extraction**

The ligation product was diluted four-fold, and 10 μL was added to 100 μL of TransStbl3 Chemically Competent Cells (TransGen Biotech, CD521-02) for transformation. Subsequently, 1 mL of SOC medium was added to the culture, shaken for two hours, transferred to 20 mL LB medium containing 50 μg/mL ampicillin and subjected to shaking for four hours, and then transferred to 300 mL LB medium containing 100 μg/mL ampicillin and shaken for 12 h to extract the AAV capsid library plasmids. All plasmids for AAV production were extracted using the Purelink HiPure Filter Maxi Kit (Thermo, K210018), according to the manufacturer’s instructions.

**AAV production and purification**

The AAV virus was generated in HEK293 cells with AAV vectors transfected using Lipofectamine LTX and Plus reagent (Invitrogen, Carlsbad, CA, USA; 94756). Our AAVs were packaged using a dual-plasmid system divided into transgenic and accessory capsid plasmids. For AAV library production, HEK293 cells were co-transfected with the AAV transgene plasmids rAAV-Cap-in-cis-lib (10 ng), pUC19 (8 μg), and the helper plasmid pRep-AAP (30 μg). Only 10 ng of the AAV capsid plasmid library was used per dish to prevent cross-packaging of variants, and 8 μg of pUC19 plasmid was included in the transfection to maintain the total amount of transfected plasmids. In addition, cells and culture medium were harvested 60 hours after transfection to limit secondary transduction of virus-producing cells, as previously described^1^. For individual rAAV production, the AAV transgene plasmid pAAV-CMV-luciferase-EGFP (8 μg) and helper plasmid (30 μg) were co-transfected into HEK293 cells per 150 mm dish. Transfected cells and culture medium were harvested after 72 hours.

The rAAV used in this study were purified as previously described^2^. Briefly, the medium was precipitated with PEG8000 (Beyotime, ST483) and collected by centrifugation. The cells were resuspended in lysis buffer (50 mM Tris-HCl pH 8.5, 150 mM NaCl, and 2 mM MgCl_2_) and subjected to three freeze-thaw cycles. This was followed by adding benzonase (Sigma, E1014-25KU) to remove DNA, and iodixanol density centrifugation was then used to purify rAAV from the crude lysate. The rAAV was then collected from the 40% layer and concentrated using PBS containing 0.001% Pluronic™ F-68 solution (Gibco, 24040032).

**AAV titration**

The AAV titration procedure below is adapted from ref^3^. AAV samples were treated with DNase I (Vazyme, DD4104) and Proteinase K (Takara, 9034) according to the manufacturer's instructions. The treated samples were diluted 1:10, 1:100, and 1:1,000 as templates for titration. The standard curves in the 20 μL reaction system were plotted using linear plasmids (digested with XhoI) of 1 × 10^12^, 1 × 10^11^, 1 × 10^10^, 1 × 10^9^, and 1 × 10^8^ copies, respectively. qPCR was performed using the AceQ Universal U + Probe Master Mix V2 (Vazyme, Q513) according to the manufacturer’s instructions. The primer and probe sequences used for GFP were as follows: GFP-F, TCCGCCACAACATCGAGGAC; GFP-R, GTAGTGGTTGTC-GGGCAGCA; and GFP-P, 6-FAM-CAGCGTGCAGCTCGCCGACC-BHQ. The reaction was performed in QuantStudio 6 Flex real-time PCR system with the following program: Step 1: 37℃ 2 min, 1 cycle; Step 2: 95℃ 10 min, 1 cycle; Step 3: 95℃ 10 s, 65℃ 45 s, 40 cycles. The copy number of the viral genomes were determined by plotting these results onto the standard curve. Raw data were exported and analyzed to calculate the copy number of the viral genomes.

**Transmission Electron Microscopy (TEM)**

As previously described^4^, AAV samples were adsorbed onto 400-μm mesh carbon-coated copper transmission electron microscopy (TEM) grids for two minutes. After washing twice with water, the grids were stained with freshly prepared 2% uranyl acetate for 30 s. After drying for a few minutes, grids were imaged using a Zeiss LEO 910 transmission electron microscope.

**Hematoxylin-eosin (H&E) staining**

Tissues were fixed with 4% formaldehyde for 1 day and washed with PBS. They were then embedded in paraffin, and 10-μm serial sections were cut and stained with H&E Staining Kit (Servicebio, G1003), according to the manufacturer’s instructions.

**Immunohistochemical (IHC) analysis**

The paraffin sections were dewaxed to water before antigen repair and then placed in 3% hydrogen peroxide solution for blocking endogenous peroxidase. The sections were incubated with 3% BSA blocking serum (Servicebio, GC305010) in PBS for 30 minutes to suppress nonspecific binding of IgG, and then incubated with rabbit polyclonal anti-KRT18/Cytokeratin18 antibody (Sino Biological, 102193-T44) diluted 1:500 in blocking solution overnight at 4°C in a humidified chamber. The slides were then washed three times with PBS and incubated with Horseradish Peroxidase conjugated goat anti-rabbit IgG (Servicebio, GB23303) at a dilution of 1:200 in PBS for 50 minutes at room temperature in the dark. The slides were washed three times with PBS, incubated with freshly prepared DAB color developing solution (Servicebio, G1212) at room temperature and restained with hematoxylin for about 3 minutes. After dewatering and sealing, the results were interpreted under a white light microscope.

**Flow cytometry**

After digestion, the single-cell suspension was treated with PE anti-human HLA-A, B, C (Biolegend, 311406) at 4℃ in the dark for 30 minutes. The cells were then washed twice with PBS and centrifuged at 50 × g for 3 minutes. Cells were resuspended in complete culture medium prior to FACS analysis. EGFP-positive (EGFP^+^) human hepatocytes and mouse hepatocytes were sorted using MoFlo XDP (Beckman Coulter) for subsequent analysis.

**AAV barcode mix preparation**

For the pooled rAAV characterization experiment, 27 variants and five controls (AAV8, AAV3B, AAVS3, LK03, and AAV9) were used to produce rAAVs packaging the plasmid pAAV-CMV-luciferase-EGFP with specific barcodes. After separate production, 32 types of AAV were mixed almost equally to form the barcode library for further validation. We yielded a total of 6.8 × 10^12^ virus particles, which were sufficient to infect three mice.

**REFERENCES**

1. Deverman, B.E. et al., 2016. Cre-dependent selection yields AAV variants for widespread gene transfer to the adult brain. Nat Biotechnol 34, 204-209.

2. Challis, R.C. et al., 2019. Systemic AAV vectors for widespread and targeted gene delivery in rodents. Nat Protoc 14, 379-414.

3. Gray, S.J. et al., 2011. Production of recombinant adeno-associated viral vectors and use in in vitro and in vivo administration. Curr Protoc Neurosci Chapter 4, Unit 4 17.

4. Horowitz, E.D. et al., 2013. Biophysical and ultrastructural characterization of adeno-associated virus capsid uncoating and genome release. J Virol 87, 2994-3002.


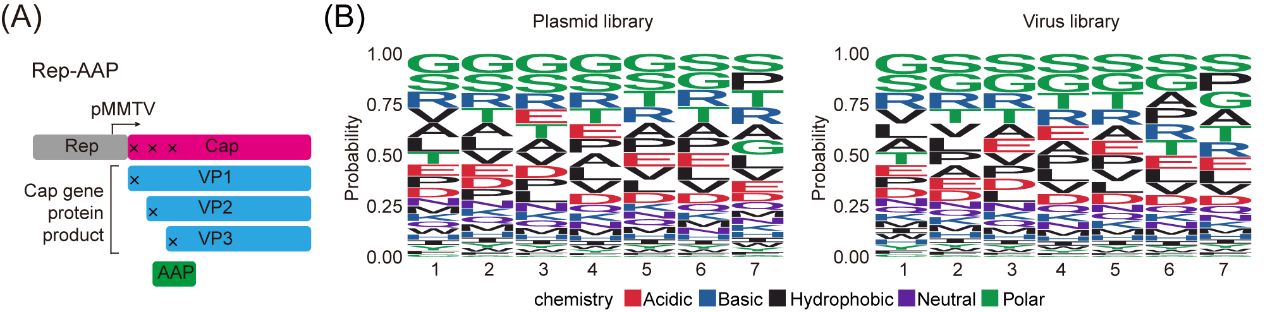


FIGURE S1. Construction of AAV9 capsid library in first round of selection. (A) The Rep-AAP AAV helper plasmid contains AAV genes and the proteins encoded by the cap gene. VP1, VP2, and VP3 capsid protein expressions are eliminated by stop codons inserted in the cap gene. (B) Amino acid distribution of plasmid library and virus library by next generation sequencing.


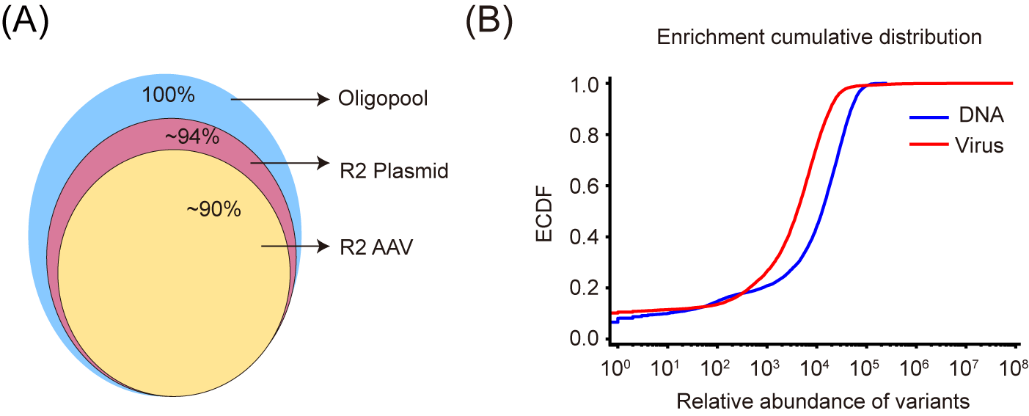


FIGURE S2. The second round of capsid library selection by synthetic pool method. (A) Overlapping bar chart shows the percentage of library overlap. (B) Empirical cumulative distribution frequency (ECDF) of the second round of DNA and virus libraries that were recovered by deep sequencing post Gibson assembly and virus production, respectively.


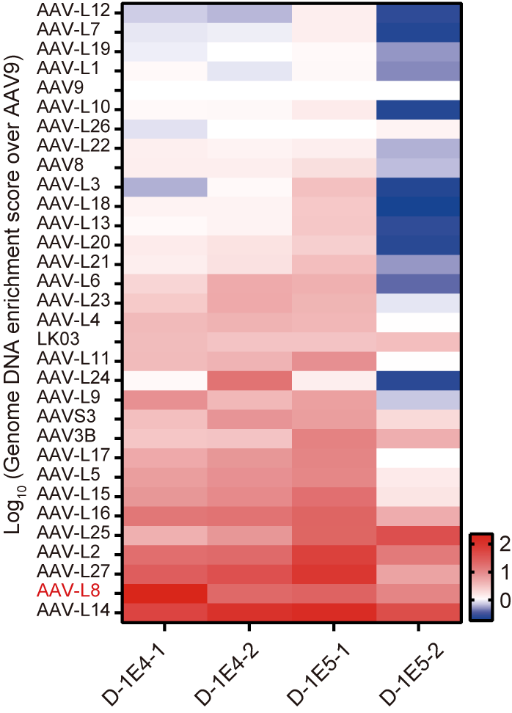


FIGURE S3. The heat map shows fold change of AAV genome DNA enrichment score over AAV9 in primary human hepatocytes (PHH). D, DNA sample. 1E4 and 1E5 represent values of multiplicity of infection (MOI).


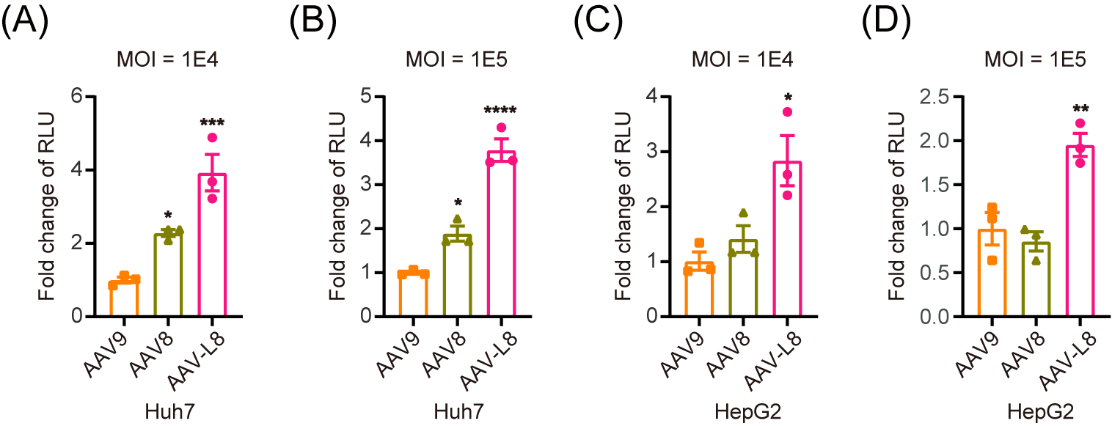


FIGURE S4. AAV-L8 transduces different human liver carcinoma cell lines more effectively. (A-B) Transduction evaluation of AAV-L8, AAV9 and AAV8 in Huh7 cell line at MOI of 1E4 (A) and 1E5 (B). (C-D) Transduction evaluation of AAV-L8, AAV9 and AAV8 in HepG2 cell line at MOI of 1E4 (A) and 1E5 (B). RLU, relative luminescence units; MOI, the multiplicity of infection. Data are presented as mean ± SEM (n = 3),*, P < 0.05; **, P < 0.01; ***, P < 0.001; ****, P < 0.0001. (one-way ANOVA using Dunnett's multiple comparison test).


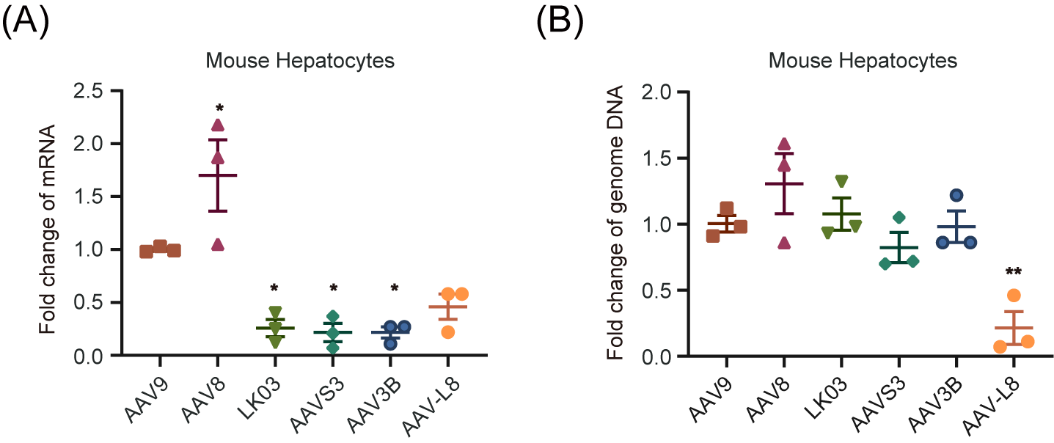


FIGURE S5. AAV-L8 shows low transduction in mouse hepatocytes. (A) Fold change of mRNA expression over AAV9 in mouse hepatocytes. (B) Fold change of transgene abundance over AAV9 in mouse hepatocytes. Data are presented as mean ± SEM (n = 3), *, P < 0.05; **, P < 0.01 (one-way ANOVA using Dunnett's multiple comparison test).

**Supplementary Tables**

**Table S1. The inserted amino acid sequences of AAV-L1~AAV-L27**

| **Variants** | **Amino acid sequence（5’→3’）** |
| --- | --- |
| AAV-L1 | PADTVEE |
| AAV-L2 | SPYTVLR |
| AAV-L3 | WGEAFNA |
| AAV-L4 | KENPSKS |
| AAV-L5 | SQKAGKG |
| AAV-L6 | SGVVQKG |
| AAV-L7 | QNPVEED |
| AAV-L8 | CSSRRSK |
| AAV-L9 | RNDQKFW |
| AAV-L10 | TPESMAE |
| AAV-L11 | WWIRSWD |
| AAV-L12 | LEAMAIE |
| AAV-L13 | NGGSRDG |
| AAV-L14 | PLSVWGC |
| AAV-L15 | MAYYKFS |
| AAV-L16 | CQSRNVC |
| AAV-L17 | WSSMASR |
| AAV-L18 | PFPTALM |
| AAV-L19 | SSVMPDG |
| AAV-L20 | RSKADQE |
| AAV-L21 | EQSPWVQ |
| AAV-L22 | VDATQPD |
| AAV-L23 | GSEKLFY |
| AAV-L24 | GNKPNVD |
| AAV-L25 | NARRRLV |
| AAV-L26 | TSEVPLV |
| AAV-L27 | DMGFRGW |

**Table S2. Barcode sequences of AAV-L1~AAV-L27**

| **Variants** | **Barcode sequence（5’→3’）** |
| --- | --- |
| AAV-L1 | CAGGTTCATACTGCCAGCTA |
| AAV-L2 | ACGCTCGACGGTTAGCGTTG |
| AAV-L3 | TTAAGCCGTTGGGTGGTTAC |
| AAV-L4 | CAAACTCCGGCAGATCGATG |
| AAV-L5 | TTTCTCGGCAATACGCACTA |
| AAV-L6 | CTATGCCGCCATTTCTAAAT |
| AAV-L7 | CGGTTATGAGAAGGGAAAGG |
| AAV-L8 | GGCAGCGTATTCTCGTTCCA |
| AAV-L9 | GCTCGTCGTGGGTCGGAGCC |
| AAV-L10 | ACTGTGCCGTCGCTTGGACG |
| AAV-L11 | TATTCTCGCGGCGAAAGTTA |
| AAV-L12 | ATGTGCTATGGTGACGAGCG |
| AAV-L13 | CCGTAAGTGAATTCGACAAT |
| AAV-L14 | TTCATAACCTCGATAGCTGG |
| AAV-L15 | TTCTTTACCAGCAGACAACG |
| AAV-L16 | CGCTGACGTCCGCAGCCCTG |
| AAV-L17 | ATGCGTACGTAAGTATGCCA |
| AAV-L18 | GTTTGGTTACCAAGATATCT |
| AAV-L19 | CATAATACAGTGTATGATCT |
| AAV-L20 | TAGGGAGTTTCCATTGCAGA |
| AAV-L21 | AATGCTGGACAGAACGAATG |
| AAV-L22 | GAACAACCGATGTGCGATTG |
| AAV-L23 | AACTATGCGTCAGATCTGGA |
| AAV-L24 | GGTTGTCATCGTCCATTGGG |
| AAV-L25 | GAGTTCTAGTCGCGGTGTCC |
| AAV-L26 | AATTCGCACCAGGCGACTCT |
| AAV-L27 | GGTATCCTAGACCATTGCGG |
| LK03 | GAAGTAAAGCTATGAGTGGC |
| AAV8 | GTACACCTGGCACGCTATGA |
| AAV9 | CGTATTCGCCAGTAGGTAAA |
| AAV3B | GTCGTGAGAATTAGATGGTG |
| AAVS3 | TGTCCATAGAACAGGAATTG |

**Table S3. The inserted amino acid sequences of AAV-M1~AAV-M50**

| **Variants** | **Amino acid sequence（5’→3’）** |
| --- | --- |
| AAV-M1 | YVSFPEP |
| AAV-M2 | VRAQWYF |
| AAV-M3 | VEFVRVM |
| AAV-M4 | RVWCSYL |
| AAV-M5 | VCTCGTR |
| AAV-M6 | QGSGGWQ |
| AAV-M7 | SGMQRSG |
| AAV-M8 | AYRVNLG |
| AAV-M9 | RLSVTSG |
| AAV-M10 | WRFKGLY |
| AAV-M11 | PEYRRSR |
| AAV-M12 | NRSGDGD |
| AAV-M13 | ITCPTHS |
| AAV-M14 | APSLDVG |
| AAV-M15 | TRQWSEF |
| AAV-M16 | MGIARGT |
| AAV-M17 | DCDCSPT |
| AAV-M18 | AHVLYRS |
| AAV-M19 | TRIQDDP |
| AAV-M20 | IMCVLFK |
| AAV-M21 | YTPKSAT |
| AAV-M22 | IRTDVID |
| AAV-M23 | RKQKGSN |
| AAV-M24 | TYTMSVP |
| AAV-M25 | WPAVPTS |
| AAV-M26 | TRVNGAT |
| AAV-M27 | LDLSEAV |
| AAV-M28 | VICPLAM |
| AAV-M29 | DKYPRQA |
| AAV-M30 | RCNWLAM |

**Table S3. The inserted amino acid sequences of AAV-M1~AAV-M50**

| **Variants** | **Amino acid sequence（5’→3’）** |
| --- | --- |
| AAV-M31 | VKTWVLG |
| AAV-M32 | NHSRSRT |
| AAV-M33 | RRASENV |
| AAV-M34 | SPRVSAA |
| AAV-M35 | GGKERWV |
| AAV-M36 | RSNIDGP |
| AAV-M37 | ACRLPLR |
| AAV-M38 | ACVLAIK |
| AAV-M39 | MASSGCP |
| AAV-M40 | KGPTPLP |
| AAV-M41 | YKPGSRD |
| AAV-M42 | RLEPCSG |
| AAV-M43 | RDRVRPS |
| AAV-M44 | VVASWIE |
| AAV-M45 | NSFSARR |
| AAV-M46 | RSPQYRT |
| AAV-M47 | LTGKGSL |
| AAV-M48 | HLVVCNP |
| AAV-M49 | YWGRECD |
| AAV-M50 | DYAAPCQ |
